# Job burnout among workers with different shift regularity: interactive factors between sleep, depression, and work environment

**DOI:** 10.3389/fpubh.2023.1131048

**Published:** 2023-08-24

**Authors:** Hyerin Gu, Jooyoung Lee, Yunjee Hwang, Jichul Kim, Somi Lee, Seog Ju Kim

**Affiliations:** ^1^Department of Psychiatry, Sungkyunkwan University College of Medicine, Samsung Medical Center, Seoul, Republic of Korea; ^2^Seoul Garden Clinic, Seoul, Republic of Korea; ^3^Deparment of Brain and Cognitive Engineering, Korea University, Seoul, Republic of Korea

**Keywords:** shift workers, burnout, job stress, depression, sleep

## Abstract

**Object:**

We investigated burnout and associated factors in non-shift workers (NSWs), shift workers with regular rotating shifts (RRSWs), and shift workers with irregular or unpredictable rotating shifts (IRSWs).

**Methods:**

In total, 5,125 adult workers (1,966 NSWs, 1,936 RRSWs, and 1,223 IRSWs) participated in an online self-reported survey. Job burnout and three dimensions thereof (exhaustion, cynicism, and professional efficacy) were assessed using the Maslach Burnout Inventory—General Survey (MBI-GS). The Center for Epidemiological Studies Depression Scale (CES-D) (depressive symptoms), Pittsburgh Sleep Quality Index (PSQI) (subjective sleep quality), and Korean Occupational Stress Scale (KOSS) (stressful job environment) were also used.

**Results:**

Both types of shift workers scored higher in terms of exhaustion, cynicism, and poor professional efficacy on the MBI-GS compared with NSWs after controlling for age and gender. IRSWs exhibited higher scores on the exhaustion and professional efficacy dimensions of the MBI-GS compared with RRSWs. After controlling for the CES-D and PSQI scores, we found no significant difference in cynicism among the groups. After controlling for the KOSS score, we found no significant difference in professional efficacy. All MBI-GS dimensional scores were correlated significantly with the CES-D, PSQI, and KOSS scores in all three groups.

**Conclusion:**

The job burnout level of rotating shift workers, especially those with irregular shifts, was higher than that of other workers. Cynicism in shift workers may be largely attributable to poor sleep or depression; the poor efficacy of shift workers may be explained by their stressful work environment.

## Introduction

Shift work is essential as services must always be available (1). Shift work is associated with personal, social, and organizational problems, including burnout, a response to prolonged (chronic) emotional and interpersonal stressors triggering extreme fatigue and loss of productivity and passion (2). Shift workers (SWs) suffer from more burnout than non-shift workers (NSWs) (3–5). The irregular sleep duration and altered bedtimes of SWs trigger burnout (4). Burnout is affected by mood (2), poor sleep (6), and the working environment (7, 8). Shift work disturbs the circadian rhythm, renders sleep poor (9), creates a depressive mood (1, 10), and emphasizes a “humble” work environment (11), in turn, triggering burnout. A comprehensive and integrative understanding of the psychological and environmental characteristics of shift workers would be helpful for investigating the factors associated with their burnout (11).

Factors associated with burnout are more common in those with irregular shifts than in those with regular shifts. Irregularly rotating SWs (IRSWs) are likely more vulnerable to burnout than regularly rotating SWs (RRSWs). IRSWs reported poorer sleep efficiency, shorter sleep duration, and poorer sleep quality compared with RRSWs (12). The greater shift intensities and shorter recovery periods of IRSWs trigger hyperarousal and insomnia (13). IRSWs more commonly experienced suicidal ideation mediated by both poor sleep and depressive mood compared with RRSWs (14). However, no study has yet compared the burnout between RRSWs and IRSWs. If the burnout is higher in IRSWs than RRSWs, it remains unclear whether sleep, mood, or the workplace environment plays the great role.

Job burnout includes three dimensions (exhaustion, cynicism, and poor professional efficacy) (15). SWs reported high levels of emotional exhaustion, cynicism, and poor professional efficacy (3–5, 16). Previous studies have reported that mood, sleep, and workplace environment could be differently associated with each individual sub-dimension of burnout (17). Both sleep disturbances and depression were correlated strongly with emotional exhaustion and cynicism, but weakly with professional efficacy (18). The occupational stress level was correlated with all three subdomains of burnout (19). However, there were some discrepancies between studies. One study reported that insomnia was associated only with emotional exhaustion, but not with cynicism or professional efficacy (20). There have also been controversies regarding how occupational stress was associated with sub-dimensions of burnout (21). These correlations may be affected by shift work or shift schedules. However, no study has yet investigated burnout of RRSWs and IRSWs in terms of these three dimensions.

This study aimed to explore burnout in NSWs, RRSWs, and IRSWs and the effects of poor sleep, mood, and the workplace environment. We hypothesized that SW, especially IRSWs, would show higher job burnout than NSWs. We also hypothesized that burnout is associated with poor sleep, depression, and a poor workplace environment.

## Methods

### Participants

A total of 6,327 workers in South Korea were recruited via an online advertisement and by a survey company (Macromill Embrain Co. Ltd.). The inclusion criteria were at least 18 years of age and having a full- or part-time job; the exclusion criterion was the inability to complete the online questionnaires. Those who worked only during daytime hours (thus not in the late evening or during the night) were defined as NSWs (normal work hours: 7 AM to 7 PM). SWs were defined as those whose working schedules rotated either regularly (RRSWs) or irregularly (IRSWs). The schedules of IRSWs were not predetermined and often changed. Regular and irregular rotations were determined depending on whether there was a fixed rule regarding the rotation of the schedules (22, 23). Shift workers whose shift schedules change according to predetermined fixed rules (e.g., 3-day shift followed by 3-night shift every week) were determined as RRSWs. Shift workers whose shift schedules change without such predetermined fixed rules regarding shift rotation were determined as IRSWs. Industry was classified into three categories [A (managers, professionals, and clerks), B (service and sales workers), and C (skilled agricultural, forestry and fishery workers, craft and related trade workers, and elementary workers)] based on the Korean Standard Classification of Occupations (KSCO) (24). Of all those enrolled, 1,202 were excluded because they were not NSWs, RRSWs, or IRSWs. There were 212 fixed evening workers, 163 fixed night workers, 816 whose schedules did not rotate, and 11 whose schedules could not be classified. Finally, data from 5,125 participants were analyzed. There was no significant difference in terms of either age or gender between the 1,202 excluded individuals (39.24 ± 10.54 years of age, 567 men and 635 women) and 5,125 included participants (37.17 ± 9.69 years of age, 2,478 men and 2,647 women). There were 1,966 NSWs (38.06 ± 9.77 years of age, 969 men and 997 women), 1,936 RRSWs (37.76 ± 9.81 years of age, 1,105 men and 831 women), and 1,223 IRSWs (34.79 ± 8.94 years of age, 404 men and 819 women). All procedures were conducted in accordance with the Declaration of Helsinki. The study protocol was approved by the Institutional Review Board of Samsung Medical Center (protocol code 2019-04-095). All participants gave written informed consent.

### Questionnaires

Depression was assessed using the short form of the Center for Epidemiological Studies Depression Scale (CES-D) (11 items). Factor analyses revealed that the short form replicates the symptom dimensions of the original CES-D, supporting the utility and structural validity of the short form across samples (25). The CES-D assesses depressive symptoms within the last week (the negatively and positively affective, somatic, and interpersonal aspects). The CES-D features a Likert-type scale with response options ranging from 1 (hardly ever or never/on <1 day during the last week) to 3 (much or most of the time/on over 5 days during the last week). A higher score indicates more severe depressive symptoms. Sleep quality was assessed using the Pittsburgh Sleep Quality Index (PSQI) (18 items), which explores subjective sleep quality, sleep latency and duration, habitual sleep efficiency, sleep disturbance, use of sleep medications, and daytime dysfunction (26). A higher score indicates poorer sleep quality. The workplace environment was assessed using the Korean Occupational Stress Scale (KOSS) (43 items), which explores stressful situations/environments (11). The KOSS consists of eight sub-factors such as physical environment, job demands, job control, interpersonal conflict, job insecurity, the organizational system, lack of rewards, and work atmosphere. The Likert-type scale responses range from 1 (strongly disagree) to 4 (strongly agree). A higher score indicates a more stressful workplace environment.

Job burnout was assessed using the Maslach Burnout Inventory—General Survey (MBI-GS) (15 items in three sub-scales: exhaustion, cynicism, and professional efficacy) (27). Exhaustion is emotional and physical fatigue caused by work stress/demands. Cynicism is a negative attitude toward work or self-distancing from work. Professional efficacy is satisfaction with one's work. The Likert scale responses range from 1 (strongly disagree) to 5 (strongly agree). A higher score indicates more burnout.

### Statistical analyses

The differences in demographic and clinical characteristics among the three groups were compared via analysis of variance. Pearson correlations were calculated to evaluate the associations among dependent variables. The Scheffe *post-hoc* analysis was performed. Analysis of covariance (ANCOVA) was employed to investigate among-group differences in burnout after controlling for age, gender, income, industry, work hours per week, depression, sleep quality, and the workplace environment. If a significant difference was apparent, further pairwise group comparisons were conducted using ANCOVA after controlling for age, gender, income, industry, work hours per week, the workplace environment, depression, and sleep quality. ANCOVA is used for examining a combination of categorical variables and a scale variable as a predictor of the dependent variables (28). The covariate can be a variable that is of interest, rather than an extraneous variable (28). In the current study, it is examined whether the combination of groups (shift work types) and covariates (sleep, depression, and workplace environment) would predict the dependent variable (burnout). *p* < 0.01 was considered indicative of statistical significance. All analyses were performed using SPSS software (version 28).

## Results

### Demographic data

The mean age of the 5,125 participants was 37.17 ± 9.69 years (range 20–69 years), and 2,647 (51.6%) were women (Table 1). The gender proportions differed significantly among the groups (*F* = 90.34, *p* < 0.001): There were significantly more women IRSWs than RRSWs and NSWs (all *p* < 0.001) and significantly more women NSWs than RRSWs (*p* < 0.001). Age also differed significantly among the groups (*F* = 49.54, *p* < 0.001), with NSWs and RRSWs being older than IRSWs (*p* < 0.001). The mean work hours per week were not significantly different among the three groups. The proportion of industry across the three groups was significantly different (*F* = 155.9, *p* < 0.001). Workers classified as B (service and sales workers) were more than C (skilled agricultural, forestry, and fishery workers, craft and related trade workers, and elementary workers) and A (managers, professionals, and clerks).

**Table 1:** Depression, poor sleep, and workplace environment-associated burnout in non-shift workers and regularly and irregularly rotating shift workers.

	**Non-shift workers (*n* = 1966)**	**Shift workers**		***F*-score**	***Post-hoc* test**
		**Regularly rotating shift workers (*****n*** = **1936)**	**Irregularly rotating shift workers (*****n*** = **1223)**		
Age (years) (Mean ± SD)	38.06 ± 9.77	37.76 ± 9.81	34.79 ± 8.94	49.54^*^	a = b < c
Gender (women) [*n* (%)]	997 (50.7)	831 (42.9)	819 (67)	90.34^*^	b < a < c
**Industry [*****n*** **(%)]**					
A	1663 (84.6)	1153 (59.6)	781 (63.9)	155.9^*^	a < c < b
B	181 (9.2)	400 (20.7)	234 (19.1)		
C	107 (5.4)	346 (17.9)	181 (14.8)		
Work hours per week (Mean ± SD)	31.36 ± 19.74	30.89 ± 20.21	31.55 ± 19.90	0.48	a=b=c
Income (Mean ± SD)	4.28 ± 0.96	4.20 ± 1.01	4.22 ± 1.00	3.44	a=b=c
CES-D score (Mean ± SD)	7.11 ± 5.83	8.43 ± 6.25	8.92 ± 6.31	39.22^*^	a < b < c
PSQI score (Mean ± SD)	6.16 ± 2.72	6.97 ± 3.00	7.04 ± 3.04	50.20^*^	a < b = c
KOSS score (Mean ± SD)	101.89 ± 13.99	104.54 ± 12.73	107.23 ± 12.34	63.72^*^	a < b < c
**MBI-GS score (Mean** ±**SD)**					
Total	39.57 ± 9.19	40.71 ± 8.55	43.15 ± 8.29	63.53^*^	a < b < c
Exhaustion	14.53 ± 4.37	15.03 ± 4.26	16.80 ± 4.14	110.64^*^	a < b < c
Cynicism	11.93 ± 3.39	12.32 ± 3.20	12.34 ± 3.20	8.68^*^	a < b = c
Professional efficacy	13.10 ± 3.03	13.36 ± 2.84	14.01 ± 2.78	37.46^*^	a < b < c

* p < 0.01. a = Non-shift workers, b = regularly rotating shift workers, c = irregularly rotating shift workers. CES-D, Center for Epidemiological Studies Depression Scale; PSQI, Pittsburgh Sleep Quality Index; KOSS, Korean Occupational Stress Scale; MBI-GS, Maslach Burnout Inventory—General Survey.

### Depression, sleep, and work environment of NSWs, RRSWs, and IRSWs

The CES-D (*F* = 39.22, *p* < 0.001) and PSQI (*F* = 63.53, *p* < 0.001) scores also differed significantly among the groups, both being lowest in the NSWs and highest in the IRSWs (all *p* < 0.001). The KOSS score differed significantly among the groups (*F* = 63.72, *p* < 0.001), with higher scores in the IRSWs than RRSWs or NSWs (all *p* < 0.001). The scores of all 8 sub-factors of KOSS differed significantly among the groups (physical environment: *F* = 6.07, *p* < 0.01; job demands: *F* = 176.47, *p* < 0.001; job control: *F* = 80.79, *p* < 0.001; interpersonal conflict: *F* = 6.07, *p* < 0.05; job insecurity: *F* = 10.10, *p* < 0.001; the organizational system: *F* = 5.63, *p* < 0.01; lack of rewards: *F* = 5.15, *p* < 0.01; work atmosphere: *F* = 15.28, *p* < 0.001). Both NSWs and RRSWs reported a more harmful physical environment, lack of job control and interpersonal conflicts, fewer job demands, and lack of rewards than IRSWs. Both RRSWs and IRSWs were less satisfied with job control, organizational system, and work atmosphere than NSWs. IRSWs reported the dissatisfaction with work atmosphere compared with RRSWs.

### Burnout of NSWs, RRSWs, and IRSWs

**Figure 1:**
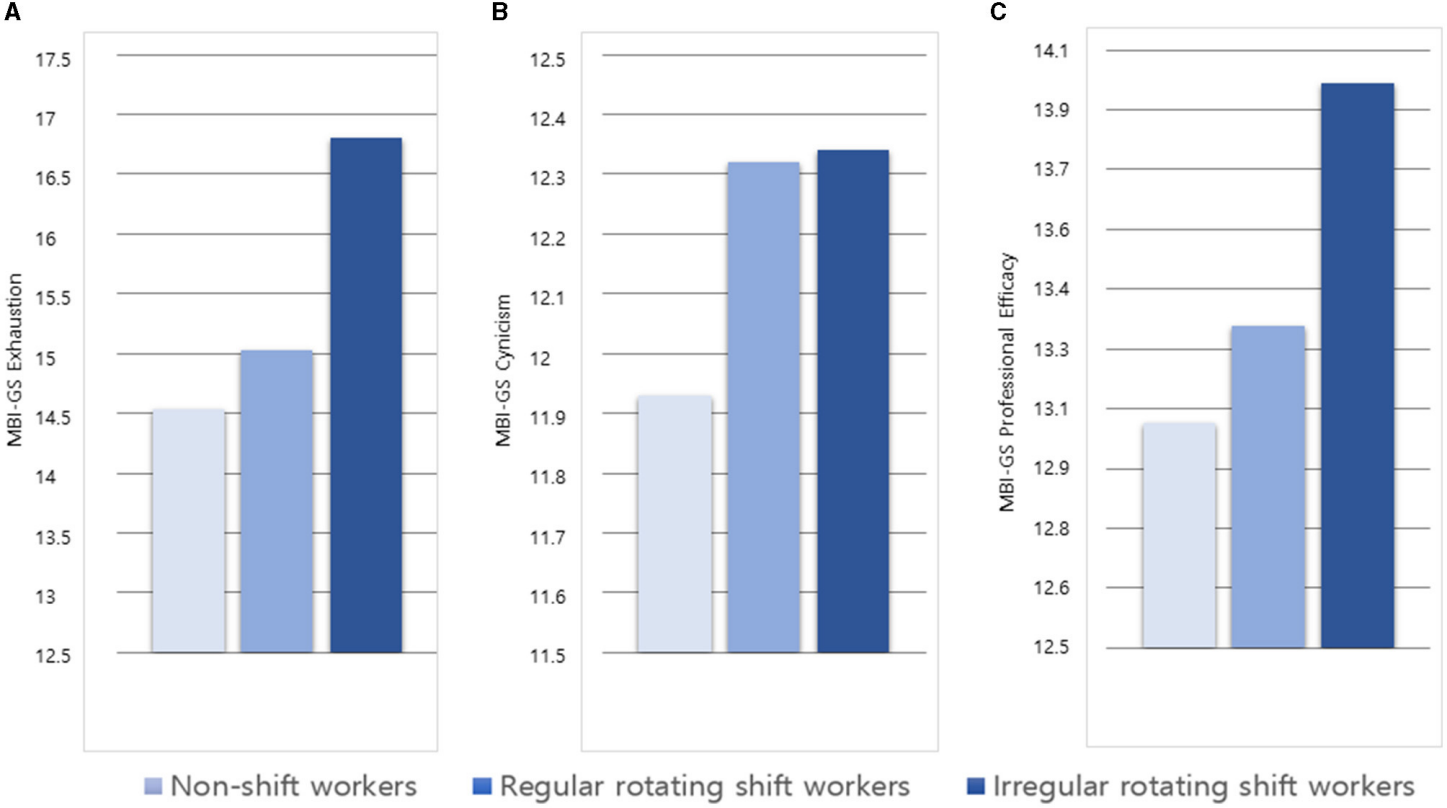
Job burnout among non-shift workers and regularly and irregularly rotating shift workers. **(A)** MBI-GS exhaustion scores of non-shift workers (light blue bar), regularly rotating shift workers (medium blue bar), and irregularly rotating shift workers (dark blue bar). **(B)** MBI-GS cynicism scores of non-shift workers, regularly rotating shift workers, and irregularly rotating shift workers. **(C)** MBI-GS Professional efficacy scores of non-shift workers, regularly rotating shift workers, and irregularly rotating shift workers.

The MBI-GS scores differed significantly among the groups (*F* = 63.53, *p* < 0.001), with higher scores in SWs than NSWs (*p* < 0.001), in IRSWs than RRSWs and NSWs (all *p* < 0.001), and in RRSWs than NSWs (*p* < 0.001). The scores of all three MBI-GS dimensions differed significantly among the groups (exhaustion, *F* = 110.64, *p* < 0.001; professional efficacy, *F* = 37.46, *p* < 0.001; cynicism, *F* = 8.68, *p* < 0.001) (Table 1, Figure 1). Both RRSWs and IRSWs reported more exhaustion and cynicism and less professional efficacy compared with NSWs. Among the SWs, IRSWs reported more exhaustion and less professional efficacy than RRSWs.

### Effects of demographic data on burnout of NSWs, RRSWs, and IRSWs

All three MBI-GS dimensions were significantly correlated with the age of NSW, RRSWs, and IRSWs. Only exhaustion and professional efficacy were significantly correlated with gender. Work hours per week and income were not significantly correlated with MBI-GS (Table 2).

**Table 2:** Correlations between demographic characteristics and job burnout.

	**Non-shift workers (*****n*** = **1,966)**			**Regularly rotating shift workers (*****n*** = **1,936)**			**Irregularly rotating shift workers (*****n*** = **1,223)**		
	**Exhaustion**	**Cynicism**	**Professional efficacy**	**Exhaustion**	**Cynicism**	**Professional efficacy**	**Exhaustion**	**Cynicism**	**Professional efficacy**
Age (years)	*r* = −0.19^*^	*r* = −0.18^*^	*r* = 0.08^*^	*r* = −0.16^*^	*r* = −0.14^*^	*r* = −0.23^*^	*r* = −0.20	*r* = −0.07^*^	*r* = −0.20^*^
Gender (female)	*r* = 0.09^*^	*r* = 0.02	*r* = −0.29^*^	*r* = 0.22^*^	*r* = 0.02	*r* = 0.14^*^	*r* = 0.23^*^	*r* = −0.04	*r* = 0.16^*^
Industry	*r* = −0.03	*r* = −0.02	*r* = 0.05^*^	*r* = 0.00	*r* = 0.01	*r* = 0.01	*r* = −0.01	*r* = 0.05	*r* = 0.02
Work hours per week	*r* = −0.01	*r* = −0.01	*r* = 0.02	*r* = 0.03	*r* = 0.01	*r* = 0.01	*r* = 0.01	*r* = −0.02	*r* = 0.00
Income	*r* = 0.01	*r* = 0.01	*r* = 0.01	*r* = 0.03	*r* = 0.02	*r* = 0.03	*r* = 0.04	*r* = −0.03	*r* = 0.00

* p < 0.01, r: correlation coefficients.

### Effects of depression, sleep, and work environment on burnout of NSWs, RRSWs, and IRSWs

All three MBI-GS dimensions were correlated significantly with the CES-D, PSQI, and KOSS scores of NSWs, RRSWs, and IRSWs (Table 3). There were no significant two-way or three-way interactions between CES-D, PSQI, and KOSS for predicting MBI-GS. The correlation of MBI-GS scores with CES-D, PSQI, and KOSS was not significantly different between groups. With correlation analysis between sub-factors of KOSS and sub-dimensions of MBI-GS across three groups, all correlations were significant except two. Only the relationship between job demand and cynicism in IRSWs and the relationship between job insecurity and exhaustion in IRSWs were not significant. As covariates (CES-D, PSQI, and KOSS) were correlated with dependent variables (MBI-GS) and had no interaction with the group on predicting dependent variables, ANCOVA could be conducted.

**Table 3:** Correlations among job burnout and depression, sleep, and the workplace environment in non-shift workers and regularly and irregularly rotating shift workers.

	**Non-shift workers (*****n*** = **1,966)**			**Regularly rotating shift workers (*****n*** = **1,936)**			**Irregularly rotating shift workers (*****n*** = **1,223)**		
	**Exhaustion**	**Cynicism**	**Professional efficacy**	**Exhaustion**	**Cynicism**	**Professional efficacy**	**Exhaustion**	**Cynicism**	**Professional efficacy**
KOSS	*r* = 0.586^*^	*r =* 0.536^*^	*r =* 0.535^*^	*r =* 0.569^*^	*r =* 0.486^*^	*r =* 0.502^*^	*r =* 0.564^*^	*r =* 0.479^*^	*r =* 0.530^*^
CES-D	*r* = 0.529^*^	*r =* 0.396^*^	*r =* 0.446^*^	*r =* 0.504^*^	*r =* 0.429^*^	*r =* 0.460^*^	*r =* 0.461^*^	*r =* 0.458^*^	*r =* 0.474^*^
PSQI	*r* = 0.300^*^	*r =* 0.162^*^	*r =* 0.200^*^	*r =* 0.338^*^	*r =* 0.238^*^	*r =* 0.260^*^	*r =* 0.312^*^	*r =* 0.198^*^	*r =* 0.215^*^

* p < 0.01. CES-D, Center for Epidemiological Studies Depression Scale; PSQI, Pittsburgh Sleep Quality Index; KOSS, Korean Occupational Stress Scale.

Group differences in job burnout were assessed after controlling for age, gender, income, industry, work hours per week, and the CES-D, PSQI, or KOSS scores (Table 4). After controlling for age and gender, income, industry, and work hours per week, the among-group differences in all three sub-dimensions of the MBI-GS remained significant (exhaustion: *F* = 66.33, *p* < 0.001; cynicism: *F* = 4.46, *p* < 0.01; professional efficacy, *F* = 15.08, *p* < 0.001).

**Table 4:** Burnout of non-shift workers and regularly and irregularly rotating shift workers after controlling for age, gender, income, industry and work hours per week, depression, poor sleep, and the workplace environment.

	**Analysis 1**	**Analysis 2**	**Analysis 3**	**Analysis 4**
**MBI-GS**				
Total	*F =* 32.87^*^	*F =* 16.09^*^	*F =* 16.98^*^	*F =* 2.81
Exhaustion	*F =* 66.33^*^	*F =* 50.46^*^	*F =* 47.28^*^	*F =* 28.31^*^
Cynicism	*F =* 4.46^*^	*F =* 1.05	*F =* 1.05	*F =* 8.81^*^
Professional efficacy	*F =* 15.08^*^	*F =* 4.95^*^	*F =* 6.48^*^	*F =* 0.34

* p < 0.01. CES-D, Center for Epidemiological Studies Depression Scale; PSQI, Pittsburgh Sleep Quality Index; MBI-GS, Maslach Burnout Inventory—General Survey; KOSS, Korean Occupational Stress Scale; MBI-GS, Maslach Burnout Inventory—General Survey.

Analysis 1: After controlling for age, gender, income, industry, and work hours per week.

Analysis 2: After controlling for age, gender, income, industry, work hours per week, and the CES-D score.

Analysis 3: After controlling for age, gender, income, industry, work hours per week, and the PSQI score.

Analysis 4: After controlling for age, gender, income, industry, work hours per week, and the KOSS score.

After additionally controlling for the CES-D score, only exhaustion and professional efficacy differed significantly among the groups (exhaustion: *F* = 50.46, *p* < 0.001; professional efficacy, *F* = 4.95, *p* < 0.01). IRSWs exhibited higher exhaustion and professional efficacy scores compared with NSWs and RRSWs. There was no significant difference in either the exhaustion or professional efficacy score between NSWs and RRSWs after additionally controlling for the CES-D score. Cynicism did not differ significantly among NSWs, RRSWs, and IRSWs after controlling for the CES-D score.

After additionally controlling for the PSQI score, exhaustion and professional efficacy differed significantly among the groups (exhaustion: *F* = 47.28, *p* < 0.001; professional efficacy, *F* = 6.48, *p* < 0.01). IRSWs showed higher exhaustion and professional efficacy scores compared with NSWs or RRSWs. No significant difference in the exhaustion or professional efficacy score between NSWs and RRSWs was apparent after additionally controlling for the PSQI scores. Cynicism did not differ significantly among NSWs, RRSWs, and IRSWs after controlling for the PSQI scores.

After additionally controlling for the KOSS scores, cynicism (*F* = 8.81, *p* < 0.001) and exhaustion (*F* = 28.31, *p* < 0.001) differed significantly among the groups. IRSWs showed higher exhaustion and cynicism scores than those of NSWs or RRSWs. There was no significant difference in the exhaustion or cynicism score between NSWs and RRSWs after additionally controlling for the KOSS scores. Professional efficacy did not differ significantly among NSWs, RRSWs, and IRSWs after controlling for the KOSS scores. Additional analyses were conducted after controlling for each one of eight KOSS sub-factors, rather than the total KOSS score. Exhaustion remained significantly different among the groups after controlling any one of eight KOSS sub-factors. The difference in cynicism between NSWs, RRSWs, and IRSWs was significant after controlling for the physical environment, interpersonal conflict, or job security, but not significant after controlling for job demands, job control, organizational system, lack of rewards, and work atmosphere. The difference in professional efficacy between NSWs, RRSWs, and IRSWs was significant after controlling for interpersonal conflict, job security, job control, organizational system, lack of rewards, and work atmosphere, while there were no significant differences after controlling for the physical environment or job demands.

## Discussion

We found a higher job burnout in rotating SWs than in the other workers, especially those with irregular schedules. Burnout was correlated with sleep disturbance, depression, and a poor workplace environment. All three burnout sub-dimension scores were higher in the IRSWs than in the other workers. Interestingly, higher-level cynicism in IRSWs was not apparent after controlling for sleep or depression, and the lower professional efficacy in IRSWs disappeared after controlling for the workplace environment. The greater exhaustion in IRSWs was independent of sleep, depression, and the workplace environment. To the best of our knowledge, this is the first study to investigate burnout and associated factors in IRSWs and RRSWs.

Our first hypothesis was that burnout would be higher in SWs than NSWs for several reasons. Circadian rhythm changes caused by shift work cause fatigue, compromise sleep quality and physical health (29), provoke anxiety, and eventually trigger job strain (3). The lack of social support and the few physical or leisure activities of night SWs were suggested to increase burnout (5). The high burnout in SWs in the current study may be attributable to the altered circadian rhythm and/or a lack of support and activity.

Of the SWs, IRSWs showed more burnout than did RRSWs. IRSWs experience chronic stress imposed by their irregular and unusual work schedules (12). Compared with other SWs, IRSWs suffer more sleep problems, associated with disruption of the light/dark rhythm that induces arousal or awakening (30), and more variability in terms of sleep duration (4). Both are associated with burnout. Poor alertness during working hours (caused by poor sleep) may increase cognitive deficit, promoting low performance (31) and poor self-esteem (14). Irregular shift schedules compromise physical and psychological health and occupational functionality. SWs, especially IRSWs, require assistance to alleviate burnout, improve job efficacy, and enhance health.

As expected, depressive symptoms, poor sleep quality, and a poor workplace environment were associated with job burnout in both NSWs and SWs. SWs experienced more occupational stress, more depressive symptoms, and poorer sleep. However, some of the increased burnout in SWs, especially increased exhaustion in IRSWs, was independent of depression, sleep, and the workplace environment.

Our study suggests that the three burnout sub-dimensions of SWs differ in terms of their associations with schedule, sleep, depression, and the workplace environment. The greater exhaustion in IRSWs compared with RRSWs remained significant after controlling for mood, sleep, and the workplace environment; the greater exhaustion in RRSWs compared with NSWs disappeared after controlling for even one of these variables. This suggests that exhaustion induced by an irregular schedule, but not a rotating regular schedule, was independent of depression, sleep, and the workplace environment. The characteristics of shift work *per se* (hours worked per day and the work period) may affect burnout (16). As the higher cynicism in IRSWs, compared with the other workers, disappeared after controlling for depression or sleep, the cynicism largely reflects poor sleep or a depressive mood. It may also be suggested that an attempt to distance oneself from work compromises mental health. In addition, although the higher cynicism in IRSWs remained significant after controlling for total KOSS scores, the group difference in cynicism disappeared after controlling for some of the workplace environments such as job demands, job control, organizational system, lack of rewards, and work atmosphere. These findings suggest that some aspects of the workplace environment may contribute to the higher cynicism of IRSWs. As the lower professional efficacy score of IRSWs remained significant after controlling for sleep or depression, professional efficacy seems to be less influenced by these factors. Unlike the other two dimensions of burnout (which are related to work overload and social conflict), poor perceived professional efficacy seems to be linked to a lack of resources (2). As the lower professional efficacy score of IRSWs disappeared after controlling for the workplace environment, professional efficacy is greatly influenced by that environment. In particular, as the lower professional efficacy score of IRSWs disappeared after controlling the physical environment or job demands, professional efficacy would be more affected by the physical environment and job demands than other factors of the workplace environment.

The factors affecting the specific dimensions of burnout in SWs may aid the formulation of strategies for reducing burnout. The work schedule of SWs would be better to be regular and predetermined to reduce burnout (32). Management of the mood and sleep problems of SWs may also reduce cynicism. Workplace mental health programs, such as sleep hygiene education, targeted mindfulness, and cognitive behavioral therapy may be helpful (33). Improvement of job demands, job control, organizational system, lack of rewards, and work atmosphere may also reduce the cynicism of SWs. Distressing the work environment of SWs, especially regarding the physical environment and job demands, would increase professional efficacy. Reducing job demands by training and support or restructuring the physical environment would be needed for improving the professional efficacy of SWs (34).

Our study has several limitations. No cross-sectional study, including our study, can address causal or temporal relationships (here, between shift work and regular work, sleep, depression, workplace environment, and burnout). Depressive symptoms and low sleep quality might be the symptoms prior to the burnout stage (35). As burnout has complex constructs having both individual stress experience and organizational work context, cross-sectional study design has definite limitations. A future longitudinal study should explore the complex multidirectional associations between burnout in SWs and related factors. Second, all data were self-reported. Objective evaluations of sleep or mood (polysomnography or structured psychiatric interviews) would have been helpful. In addition, all participants were Korean. Caution is needed in applying our findings to other cultures. Finally, the SWs of the current study worked in various fields; most previous studies on SWs focused on specific occupations (healthcare workers, police officers, and industrial workers) (1). Although the findings of this research assist such workers, our findings are applicable to more general SWs.

In conclusion, we found that the burnout of SWs, especially IRSWs, was higher than that of NSWs. Poor sleep, depressive symptoms, and poor workplace environments were associated with burnout, but their influence on the association between shift work and burnout may differ by the different burnout sub-dimensions. Exhaustion of SWs may be attributable to shift work *per se*, especially irregular shift work. The higher cynicism of IRSWs may reflect sleep or mood problems, and the lower professional efficacy of IRSWs may be attributed to the workplace environment.

## Data availability statement

The raw data supporting the conclusions of this article will be made available by the authors, without undue reservation.

## Ethics statement

The studies involving humans were approved by the Institutional Review Board of Samsung Medical Center (protocol code 2019-04-095). The studies were conducted in accordance with the local legislation and institutional requirements. The participants provided their written informed consent to participate in this study. Written informed consent was obtained from the individual(s) for the publication of any potentially identifiable images or data included in this article.

## Author contributions

HG, JL, and SK: conceptualization. SK: supervision, resources, investigation, and funding acquisition. HG: visualization and writing—original draft preparation. HG and SK: formal analysis. JL, SL, and SK: validation and writing—review and editing. HG, YH, and JK: methodology and data curation. All authors contributed to the article and approved the submitted version.
